# Editorial: Early development of sound processing in the service of speech and music perception

**DOI:** 10.3389/fnhum.2024.1471445

**Published:** 2024-08-13

**Authors:** Judit Gervain, Teija Kujala, Marcela Peña, Laurel J. Trainor, István Winkler

**Affiliations:** ^1^Integrative Neuroscience and Cognition Center, CNRS & Université Paris Cité, Paris, France; ^2^Department of Social and Developmental Psychology, University of Padua, Padua, Italy; ^3^Cognitive Brain Research Unit & Centre of Excellence in Music, Mind, Body, and Brain, Department of Psychology, Faculty of Medicine, University of Helsinki, Helsinki, Finland; ^4^School of Psychology, Cognitive Neuroscience Lab, Pontificia Universidad Católica de Chile, Santiago, Chile; ^5^National Center for Artificial Intelligence, CENIA FB210017, Basal ANID, Santiago, Chile; ^6^Psychology, Neuroscience & Behaviour and the LIVELab, McMaster University, Hamilton, ON, Canada; ^7^Institute of Cognitive Neuroscience and Psychology, HUN-REN Research Centre for Natural Sciences, Budapest, Hungary

**Keywords:** early development, auditory perception, speech perception, music perception, infants, toddlers

Speech and music are the two structurally most complex auditory signals that infants typically encounter. Yet, even in the presence of multiple sound streams, healthy human infants display signs of music perception, such as moving to a musical rhythm as early as a few months of life and by 18–24 months of age they understand many simple sentences. Extracting and analyzing speech and music at such an early age proves that many of the higher-order processing capabilities, such as regularity detection, auditory stream segregation, statistical learning, and rhythm processing are already present at birth or develop quite early during infancy. Understanding how the infant brain processes sound not only provides insights into the neural and processing prerequisites of speech and music perception and—compared to adults—a simpler model of the mechanics of these functions, but it is also essential for developing early interventions for atypically developing infants, such as designing training protocols for infants at risk of auditory developmental deficits.

The present Research Topic of papers consists of empirical studies and reviews examining the functioning of auditory information processing necessary for speech and language perception in infancy. The 11 papers collected can be sorted into four main research area: (1) higher-order auditory functions supporting speech and music processing, (2) rhythm, (3) speech processing in infants, and (4) predicting the quality of the outcome of language acquisition from data collected from prelingual infants and their families. Here we will summarize the main conclusions of the papers in the Research Topic in relation to the state-of-the-art of their larger research fields.

## Higher-order auditory functions supporting speech and music processing

In developing their capabilities to extract information from speech and music, infants rely on general auditory functions supporting in-depth analysis of sound sequences. Because multiple sound sources are active in real-life situations, detailed analysis of any sound sequence must be preceded by separating it from the rest of the sounds (auditory stream segregation). Calcus' review of the development of auditory scene analysis from infancy to adulthood concludes that while some of the processes segregating auditory streams by sequential as well as by simultaneous cues are present at birth, these functions have long developmental trajectories.

Both speech and music abound in dependencies between nonadjacent elements of sound sequences. Mueller et al., showed that 3-year-old children can learn the dependency between two pure tones separated by a random third one. Further, the size of the electroencephalographic response to infrequent tones violating the dependency rule correlates with that to the response to deviance in tone intensity.

## Rhythm processing in infants

The majority of previous studies on infants' rhythm processing have focused on auditory stimuli, but in real environments, rhythms occur in multimodal contexts. The two studies on rhythm in the present Research Topic targeted different multimodal interactions. Cirelli et al. explored whether infants attend (look longer) to videos where a hand taps are at the same tempo as an auditory rhythm compared to when there is a mismatch in tempo. They found no evidence for this, but exploratory analyses suggested there are complex interactions between pitch, tempo and rhythmic complexity that affect infants' attention to audio-visual synchrony. Boll-Avetisyan et al. explored auditory-motor interactions in the context of duration, pitch and intensity cues to rhythmic grouping structure in isochronous speech-syllable streams. While, as predicted, duration cues led to the greatest amount of infant rhythmic movement, they found the opposite of their prediction that infants who engaged in more rhythmic movement would show better speech segmentation. This leads to questions about how infant rhythmic movements relate to language learning. Both studies are intriguing in demonstrating the complexity of multi-modal rhythm processing and open the door for more research in this important area.

## Speech processing in infants

Infancy includes rapid development of skills needed for understanding speech: identification of phonemes, integrating them to wordforms, and detecting morpheme and word relations. Whereas it has been shown earlier that already neonates can distinguish between phonemes, the study of Hegde et al. showed that the developmental trajectories for acquiring vowels and consonants may differ. They found that between 6 and 10 months, infants have different trajectories in the perceptual weight of temporal acoustic cues for consonant and vowel processing. Piot et al., in turn, found that 9-month-old infants are even sensitive to regularities of phoneme contrasts, which are difficult to discriminate at an early age. The intensive period of phoneme learning during the 1^st^ year of life is followed by, and partly overlapping with, vocabulary acquisition. Ylinen et al. assessed in 1-year-olds the processing of word forms right after learning them compared to words learned earlier. They found that newly learned and earlier learned words appear to be processed similarly. A less studied but potentially important stage of language learning is the fetal stage. The study of Gorna-Careta et al. found that neonates of bilingual compared to monolingual mothers are more sensitive to a wider range of speech frequencies, possibly due to the greater speech signal complexity of bilingual mothers.

These early steps of speech analysis are followed by the acquisition of linguistic meaning, of which Forgács presents an interesting review and proposes that the access to meaning is possible thanks to the development of the theory of mind ability, providing some of the few available infant brain data in the field. This article opens the door to provoking questions such as how the attribution of mental beliefs arises in the absence of meaning.

## Predicting language developmental outcomes

In the last decade, individual predictors of language outcomes have received increasing attention, in part in an attempt to provide early and efficient interventions for children at risk for language delay and disorders, as these have a strong negative impact on academic and later professional outcomes. In this vein, Ortiz-Barajas showed that newborn infants' differential theta oscillations discriminating between their native language and a rhythmically different unfamiliar language predict infants' vocabulary sizes at 12 and 18 months. Non-linguistic abilities also contribute to language development. Balázs et al. found that apart from gender and gestational age, already known predictors of language development, temperament also contributed to linguistic abilities in infants and toddlers, with more sociable and responsive children showing better language skills.

The current Research Topic of papers demonstrates that while studying the roots and early development of speech and music perception requires much ingenuity and often poses methodological challenges, there is substantial progress in the field through the efforts of an ever-widening circle of researchers world-wide.

## Author contributions

JG: Writing – original draft, Writing – review & editing. TK: Writing – original draft, Writing – review & editing. MP: Writing – original draft, Writing – review & editing. LT: Writing – original draft, Writing – review & editing. IW: Conceptualization, Writing – original draft, Writing – review & editing.

