# Corrigendum: Editorial: Early development of sound processing in the service of speech and music perception

**DOI:** 10.3389/fnhum.2024.1540202

**Published:** 2024-12-23

**Authors:** Judit Gervain, Teija Kujala, Marcela Peña, Laurel J. Trainor, István Winkler

**Affiliations:** ^1^Integrative Neuroscience and Cognition Center, CNRS & Université Paris Cité, Paris, France; ^2^Department of Social and Developmental Psychology, University of Padua, Padua, Italy; ^3^Cognitive Brain Research Unit & Centre of Excellence in Music, Mind, Body, and Brain, Department of Psychology, Faculty of Medicine, University of Helsinki, Helsinki, Finland; ^4^School of Psychology, Cognitive Neuroscience Lab, Pontificia Universidad Católica de Chile, Santiago, Chile; ^5^National Center for Artificial Intelligence, CENIA FB210017, Basal ANID, Santiago, Chile; ^6^Psychology, Neuroscience & Behaviour and the LIVELab, McMaster University, Hamilton, ON, Canada; ^7^Institute of Cognitive Neuroscience and Psychology, HUN-REN Research Centre for Natural Sciences, Budapest, Hungary

**Keywords:** early development, auditory perception, speech perception, music perception, infants, toddlers

In the published article, there was an error in the Funding statement. Instead of: “The author(s) declare that no financial support was received for the research, authorship, and/or publication of this article.” The correct Funding statement appears below.

